# EXPOSURE AND EMOTIONAL REACTIVITY TO DAILY STRESS IN SAME-SEX AND DIFFERENT-SEX MARRIAGES

**DOI:** 10.1093/geroni/igz038.2935

**Published:** 2019-11-08

**Authors:** Michael Garcia, Rachel Donnelly, Debra Umberson

**Affiliations:** 1 Population Research Center, Austin, Texas, United States; 2 Population Research Center, The University of Texas at Austin, Austin, Texas, United States

## Abstract

Recent work exploring links between stress processes and well-being within marriage suggest that women may be at an increased risk for exposure and emotional reactivity to daily stress. However, studies have focused primarily on heterosexual couples, raising questions concerning whether and how these gendered patterns might unfold differently for men and women in same-sex marriages. In the present study, we analyze 10 days of dyadic diary data from 756 midlife men and women in 378 gay, lesbian, and heterosexual marriages to consider how exposure and emotional reactivity to daily stress may differ across union types. We find that women are exposed to more daily stressors than men and that this exposure is especially detrimental to the well-being of women in different-sex marriages. These findings highlight the need to include same-sex couples when exploring gendered linkages between daily stress processes and well-being within marriage.

